# Effect of Cd^2+^ Substitution on Structural–Magnetic and Dielectric Properties of Ni–Cu–Zn Spinel Ferrite Nanomaterials by Sol–Gel

**DOI:** 10.3390/molecules28166110

**Published:** 2023-08-17

**Authors:** Hu Yang, Xingxing Yang, Jinpei Lin, Fang Yang, Yun He, Qing Lin

**Affiliations:** 1 College of Biomedical Information and Engineering, Hainan Medical University, Haikou 571199, China; 2 Guangxi Key Laboratory of Nuclear Physics and Nuclear Technology, College of Physics and Technology, Guangxi Normal University, Guilin 541004, China; 3 Department of Civil Engineering, Jiangxi Water Resources Institute, Nanchang 330013, China

**Keywords:** Ni–Cu–Zn spinel ferrite, SEM, dielectric loss, Rietveld refinement

## Abstract

Cd*_x_*Ni_0.5−*x*_Cu_0.2_Zn_0.3_Fe_2_O_4_ (0 ≤ *x* ≤ 0.50) ferrite with a spinel structure was prepared using the sol–gel self-propagation method. The effects of Cd^2+^ doping on the structure, morphology, dielectric, and magnetic properties of Ni–Cu–Zn ferrite were examined using XRD, SEM, EDX, FTIR, MPMS, and dielectric tests. The cubic spinel structure was verified by XRD and FTIR analyses. The crystallite size and particle size information of the samples were obtained with XRD and SEM analysis. The sample particle size belonged to a class of nanoscale materials with a particle size range of 1–100 nm. The minor difference between the grain size and particle size indicated that the sample nanoparticles were composed of numerous microcrystals. The EDX spectra indicated that the samples contained all stoichiometric elements. MPMS was used to measure the hysteresis lines of the samples. According to the hysteresis line, the saturation magnetization intensity (M_s_), coercivity (H_c_), and magnetic moment (μ_B_) of the sample increased and then decreased with the increase in cadmium concentration. The magnetization strength (M_s_) is between 4–67 emu/g, and the coercivity (H_c_) is between 9–46 Oe. The curves of the real part of the dielectric constant (ε′), the imaginary part of the dielectric constant (ε″), and the loss factor (tanδ) with frequency were measured in the frequency range 100 Hz–100 kHz by means of an impedance analyzer. The complex modulus spectrum was analyzed to understand the dynamics of the conduction process.

## 1. Introduction

Ferrite materials possessing spinel structures are crucial for basic disciplinary research, particularly in the study of the underlying relationship between crystal structure and magnetic effects [1,2]. The spinel-structured ferrite AFe_2_O_4_ has superior structural, magnetic, and dielectric properties [3,4,5]. It also has potential applications in areas such as high-density data storage, ferromagnetic fluids, color imaging, high-frequency devices, and drug delivery systems [4,6]. Spinel-type ferrites are prepared by the sol–gel self-propagation method [6,7,8,9], spray pyrolysis [10,11], co-precipitation [3,12,13,14,15], and microemulsion methods [16,17,18,19]. The sol–gel self-propagation process is superior for producing high-purity ferrite materials due to its simple operation, low cost, low energy consumption, and crystallized powder uniformity [10,20,21,22]. The structure and properties of spinel-type ferrite nanomaterials are influenced by the method of preparation [7,9]. Spinel ferrite has an Fd-3m space group [1]; oxygen ions produce a face-centered cubic (fcc) tightly-packed arrangement [8]. Metal ions occupy tetrahedral (A) or octahedral (B) positions, with oxygen ions as the backbone [4,23,24]. The variation of different ions in the tetrahedral or octahedral positions can alter the structural, electrical, magnetic, and catalytic properties of spinel-type ferrite nanomaterials [17,25,26]. Rahmayeni et al. [27] synthesized CuFe_2_O_4_/activated carbon (CuFe/AC) composites from activated carbon extracted from oil palm waste using green shellfish leaf extract as a biocovering agent, which showed a photocatalytic degradation activity up to 92.10% of rhodamine B under 2 h of light. 0.2CuFe/0.8AC composites can be reused by maintaining their high dye-degradation rate. Sandhya Mishra et al. [28] produced spinel nickel ferrite (SNFO) 2D flakes using a co-precipitation technique. The degradation of tetracycline hydrochloride (TCH) antibiotic by nickel spinel ferrite was investigated and compared with zinc spinel ferrite (SZFO). The microwave degradation was completed within 15 min; the degradation performance was about 90% for the SNFO catalyst and 86% for the SZFO catalyst. Faisal Suleiman Mustafa et al. [29] synthesized Ag-doped Ni_0.5_Zn_0.5_Fe_2_O_4_ photocatalyst (Ag-D-NZF) for the degradation of metronidazole antibiotics and bacteria-contaminated water. The degradation of metronidazole under UV irradiation of Ag-doped Ni_0.5_Zn_0.5_Fe_2_O_4_ reached 99.9%.

Ni–Cu–Zn spinel ferrite nanomaterials have a wide range of applications in both high-frequency and magnetic memory devices. The most common material for producing multilayer chip inductors (MLCIs) is Ni–Cu–Zn spinel ferrite [18,19]. MLCIs are made of alternating layers of silver electrodes and spinel ferrites. A key strategy for reducing the number of MLCI layers and increasing efficiency is to further reduce the size of the electronics. By modifying the metal ion ratio and/or replacing alternative metal ions, the Ni–Cu–Zn spinel ferrite produced has high resistivity, low coercivity, and a low dielectric constant [19]. Juan Carlos Aphesteguy et al. [20] prepared Ni_0.35_Cu_0.15_Zn_0.5_Fe_2_O_4_ magnetic nanoparticles using the sol–gel combustion method. Mössbauer spectroscopy results indicated the presence of a paramagnetic doublet state in the samples, and the coercivity field of Ni_0.35_Cu_0.15_Zn_0.5_Fe_2_O_4_ magnetic nanoparticles decreased with Fe^2+^ substitution at low temperature (260 Oe). Qing Lin et al. [21] prepared Ni_0.6_Cu_0.2_Zn_0.2_Ce*_x_*Fe_2−*x*_O_4_ (0 ≤ *x* ≤ 0.85) using the sol–gel method. XRD spectral analysis indicated that the average grain size of the samples decreased with increasing Ce^3+^ substitution, and the lattice parameters varied with the *x* content. The Mössbuaer room-temperature spectra confirm that the [Fe^3+^-O^2−^-Fe^3+^] superexchange interactions are reduced as a result of Ce^2+^ substitution. Substitution of divalent or trivalent metal ions such as Ni-Cu-Zn-Fe-V [22], Ni-Cu-Zn-Fe-Gd [23], and Ni-Cu-Zn-Fe-La-Y [19] for Fe^3+^ in Ni–Cu–Zn spinel ferrites has been frequently reported to modify the structural, electrical, and magnetic characteristics of these materials. However, studies on the replacement of Ni/Cu/Zn ions in Ni–Cu–Zn spinel ferrite by divalent or trivalent transition metal ions to obtain quaternary materials are relatively limited. The doping of cadmium ions significantly impacts the structural, magnetic, and electrical properties of the material [1,10] due to the large radius of cadmium ions and their preference for a tetrahedral environment. Therefore, utilizing isolator-doped Ni–Cu–Zn spinel ferrite will be of significant interest.

In this study, we innovatively doped Ni–Cu–Zn spinel ferrite with divalent excess metal particles (Cd^2+^) and investigated the influence of Cd^2+^ doping on the lattice structure of Ni–Cu–Zn spinel prepared with the sol–gel method. Furthermore, we discussed the effects of Cd^2+^ doping on the morphology, magnetic properties, and dielectric properties of Ni–Cu–Zn spinel ferrite.

## 2. Results and Discussion

### 2.1. XRD Analysis

**Figure 1:**
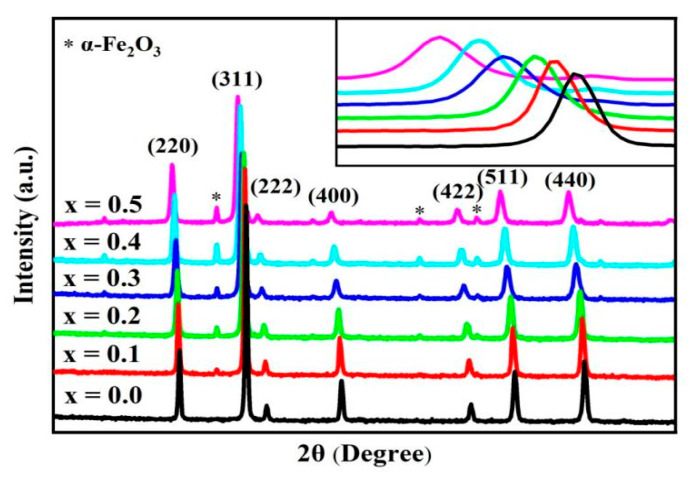
XRD patterns of Cd*_x_*Ni_0.5−*x*_Cu_0.2_Zn_0.3_Fe_2_O_4_ (0 ≤ *x* ≤ 0.50, in the steps 0.10). The peaks associated with impurity hematite hexagonal phase a-Fe_2_O_3_ are marked with asterisks.

Figure 1 illustrates the XRD patterns of the spinel ferrite system Cd*_x_*Ni_0.5−*x*_Cu_0.2_Zn_0.3_Fe_2_O_4_ (0 ≤ *x* ≤ 0.50, in steps of 0.10). The Miller index (hkl) of the spinel phase is marked in the figure, corresponding to the (220), (311), (222), (400), (422), (511), and (400) crystal planes. The XRD patterns were analyzed using Jade 5 software, revealing the formation of a cubic spinite structure with space group Fd-3m, which was in accordance with the JCPDS standard card number 74-0748. Moreover, we observed a second phase apart from the sample (*x* = 0). The second phase was a peak of the hematite hexagonal phase α-Fe_2_O_3_ (JCPDS # 86-0550), with space group R-3c and lattice parameters a = b = 5.03 Å and c = 13.73 Å. M. H. Nasr et al. used Ni-doped Fe–Cd spinel nanoferrite [8], which also produced the secondary phase -Fe_2_O_3_ as the Ni doping increased. Vivek Verma et al. [9] synthesized L_i0.35_Cd_0.3_Fe_2.35_O_4_ lithium–cadmium ferrite with secondary-phase α-Fe_2_O_3_ generation using a hybridized citrate gel precursor technique in a different pH medium. Munish Gupta et al. [14] prepared cadmium ferrite (CdLi*_x_*Fe_(2−*x*)_O_(4−*x*)_); 0 ≤ *x* ≤ 0.50) using ammonium hydroxide as a co-precipitant, and also observed the presence of Fe_2_O_3_. This result shows that the solid solution limit of Cd-doped Ni–Cu–Zn spinel ferrite is less than *x* = 0.1. XRD patterns can be derived for the lattice constant (a), grain size (D), and X-ray density (ρ_x_).

**Figure 2:**
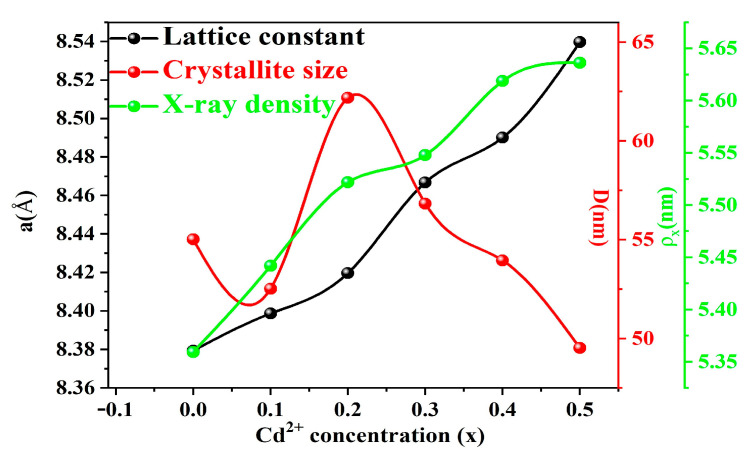
Variation of the sample lattice constant, grain size, and X-ray density according to the amount of Cd^2+^ doping.

Figure 2 illustrates the nonlinear relationship between the sample’s lattice constant (a), grain size (D), and X-ray density (ρ_x_) and the cadmium concentration. 

The inset image in Figure 1 illustrates how the main peak of spinel ferrite (311) changes to a lower angle with the increase in cadmium doping.

This phenomenon can be explained by Bragg’s law and formula in the following ways:

(1) $$d=\frac{n\lambda }{2\sin\;\theta }$$

where d is the crystal plane spacing, λ is the X-ray wavelength, and θ is the Bragg angle. The doping of cadmium ions into Ni–Cu–Zn spinel ferrite increases the ferrite crystal plane spacing (d) because cadmium has a higher ionic radius (0.97 Å) than nickel (0.69 Å). This eventually results in a shift from the Bragg angle to a smaller angle. Additionally, Figure 2 shows a positive correlation between increasing Cd content and the change in both the X-ray density (ρ_x_) and the cell parameters of the samples. The X-ray density can be estimated using the lattice constant and Equation (2).

(2) $$\rho_{x}=\frac{ZM}{{Na}^{3}}$$

where M is the molecular weight of the sample, N is the Avogadro constant (6.023 × 10^23^ mol^−1^), a is the lattice constant, and Z is the number of molecules in each cell. The equation shows that the ρ_x_ value is inversely related to a^3^. However, because the ρ_x_ value is positively related to M, and the sample’s molecular weight increases with the increase in Cd (Cd molecular weight is larger than Ni ions), the overall ρ_x_ value and the cell parameters of the sample are positively correlated.

**Figure 3:**
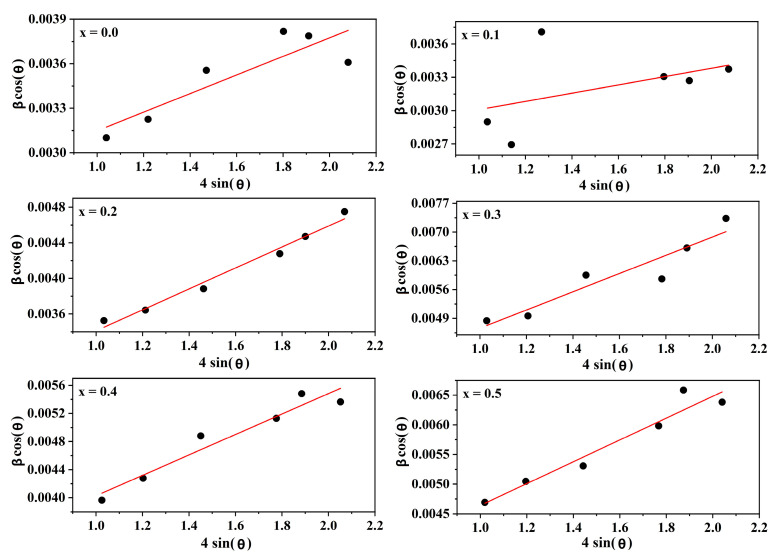
Williamson–Hall plot of Cd*_x_*Ni_0.5−*x*_Cu_0.2_Zn_0.3_Fe_2_O_4_ (0 ≤ *x* ≤ 0.50, in steps of 0.10).

As shown in Figure 3, Sample grain size and lattice strain can be estimated by linearly fitting the Williamson–Hall formula with 4sin(θ) as the horizontal coordinate and βcos(θ) as the vertical coordinate. The slope of the plot represents the strain (ε), while the intercept on the y-axis determines the grain size. 

In Figure 3, a linear fit to the Williamson–Hall equation may be used to estimate the grain size and lattice strain. Table 1 presents the structural features of Cd^2+^-doped Ni–Cu–Zn spinel ferrite nanomaterials. In this study, the Williamson–Hall formula is used to determine the induced strain brought on by lattice defects and distortions in the nanocrystals.

The grain size and lattice strain of the Cd*_x_*Ni_0.5−*x*_Cu_0.2_Zn_0.3_Fe_2_O_4_ sample were estimated using the deformation model (UDM) given in Equation (3) [8]. The Williamson–Hall equation for determining the average grain size and lattice strain is as follows:

(3) $$\beta_{T}\cos\;\theta =\varepsilon \left(4\sin\;\theta \right)+\frac{K\cdot \lambda }{D}$$

where D is the grain size, λ is the X-ray wavelength, K is a constant (0.9), and β is the half-peak width.

### 2.2. XRD Rietveld Refinement

**Figure 4:**
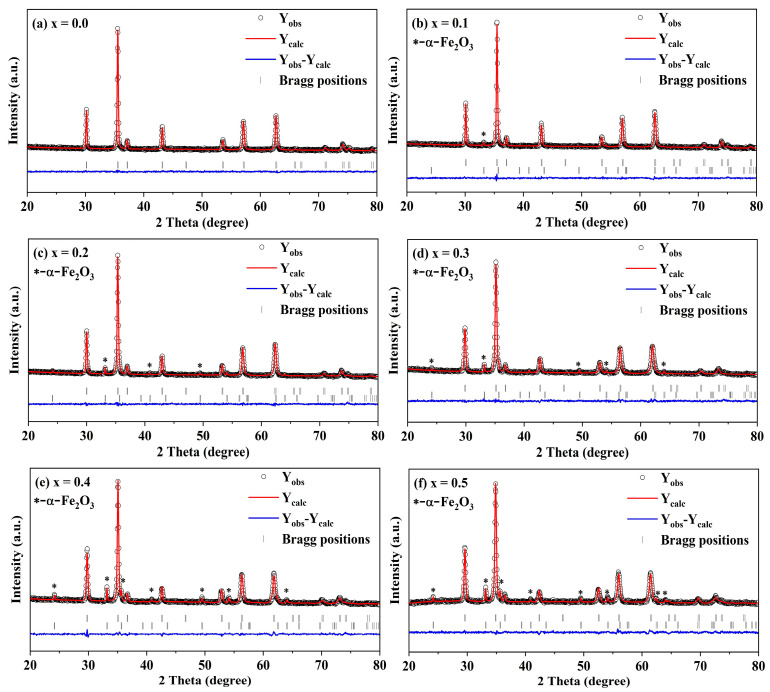
XRD mapping refined with Rietveld. Observed (symbols) and calculated (line) patterns for all samples. Vertical bars indicate positions of Bragg reflection for the existing phases. The difference between calculated and observed intensities is shown in the bottom. The peaks associated with impurity hematite hexagonal phase a-Fe_2_O_3_ are marked with asterisks.

The XRD of the spinel ferrite system Cd*_x_*Ni_0.5−*x*_Cu_0.2_Zn_0.3_Fe_2_O_4_ (0 ≤ *x* ≤ 0.50, in steps of 0.10) was subjected to Rietveld refinement in order to further investigate the crystal structure of the samples, as shown in Figure 4.The refinement results indicate that samples have an Fd-3m space group with increasing lattice constants and an increasing trend in the volume fraction of α-Fe_2_O_3_. The lattice constants of the samples obtained after the Rietveld refinement are very similar to those obtained by the XRD fit. The ionic radii of the doped ions can account for the increase in lattice constant with increased Cd concentration. Moreover, the significant difference in ionic radii between Cd^2+^ (0.97 Å) and Ni^2+^ (0.69 Å) may be the primary cause of this phenomenon. The R-factor, refinement parameters, and goodness-of-fit are reported in Table 2. Harpreet Kaur et al. [7] used Zn^2+^ and Cd^2+^-doped cobalt ferrite, and the experimental results found that the lattice constant of the sample becomes larger after doping. The explanation is that the ionic radii of Zn^2+^ (0.74 Å) and Cd^2+^ (0.97 Å) are larger than that of Co^2+^ (0.70 Å), which is in agreement with experimental results of the present work. M. H. Nasr et al. [8] employed Ni^2+^-doped cadmium ferrite and found that the lattice constant keeps getting smaller with increasing Ni content. This experimental result is corroborated by our experimental results.

Table 3 shows the tetrahedral bond length (A-O), octahedral bond length (B-O), hopping length at the A-site (L_A_), hopping length at the B-site (L_B_), A-site cation radius, and B-site cation radius. The tetrahedral bond length and octahedral bond length keep increasing with the increase in Cd doping. This indicates that Cd^2+^ ions diffuse in the spinel structure tetrahedra and cause some ions in the original tetrahedra to diffuse into the octahedral. Muhammad Imran Arshad et al. used La^3+^-doped Mg-Cd-Cu ferrite; the experimental results showed an increase in both tetrahedral bond lengths (A-O) and octahedral (B-O) bond lengths and concluded that the increase in octahedral bond lengths is due to the diffusion of La^3+^ across the octahedra simultaneously, resulting in the diffusion of some of the ions from the original octahedra into the tetrahedra. These experimental conclusions are applicable to the present work [24].

The distance between the A-site cations (hopping length L_A_) and the distance between the B-site cations (hopping length L_B_) was estimated using the following equations:

(4) $$L_{A}=\frac{a\times \sqrt{3}}{4}$$

(5) $$L_{B}=\frac{a\times \sqrt{2}}{4}$$

It is clear from Equations (4) and (5) that the A-position hopping length (L_A_) and the B-position hopping length (L_B_) are directly related to the lattice constants. The hopping lengths L_A_ and L_B_ of A- and B-positions keep increasing with the increase in Cd incorporation. This can be explained by the cell expansion due to the increase in site radius, caused by the entry of larger Cd^2+^ ions into the tetrahedral lattice and smaller ions into the octahedral lattice. The experimental findings are consistent with the cell and bond length variation patterns obtained by M.H. Nasr et al. [8] using Ni^2+^ ion-doped Cd ferrite. The following equations can be used to determine the radius of the A-site cation (r_A_) and the radius of the B-site cation (r_B_):

(6) $$L_{A}=(A-O)-r(O^{-2})$$

(7) $$L_{B}=(B-O)-r(O^{-2})$$

### 2.3. SEM Analysis

**Figure 5:**
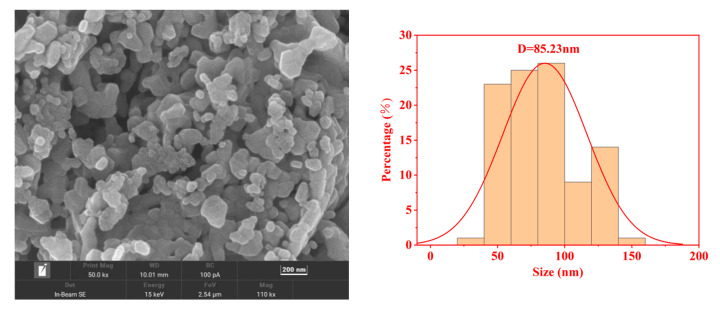
SEM images and particle-size statistics of Cd*_x_*Ni_0.5−*x*_Cu_0.2_Zn_0.3_Fe_2_O_4_ samples.

SEM tests were conducted on representative samples where *x* = 0.1 in order to examine the surface morphology of spinel-structured ferrite. Figure 5 indicates that the surface morphology of the sample where *x* = 0.1 is inhomogeneous, spherical, and highly agglomerated. The agglomeration phenomenon of the sample particles is a natural consequence of the sol–gel self-propagation method of synthesis [6]. The particle distribution of the samples was plotted. The mean particle size was determined by fitting the histogram of the particle distribution to a normal distribution function. The average grain size obtained statistically in SEM is approximately 85.23 nm, which is larger than the grain size estimated by the Williamson–Hall formula. This indicates that each nanoparticle in the synthesized sample consists of numerous microcrystals. Harpreet Kaur. et al. [7] prepared Cd-doped Co ferrite samples using the sol–gel method. In addition, the experimental results indicated that the particle size (D_SEM_) obtained by scanning electron microscopy statistics of the samples was 58.83 nm, and the microcrystal size (D_W-H_) estimated by the Williamson–Hall formula was 47.79 nm. This result is consistent with the results of the present work.

**Figure 6:**
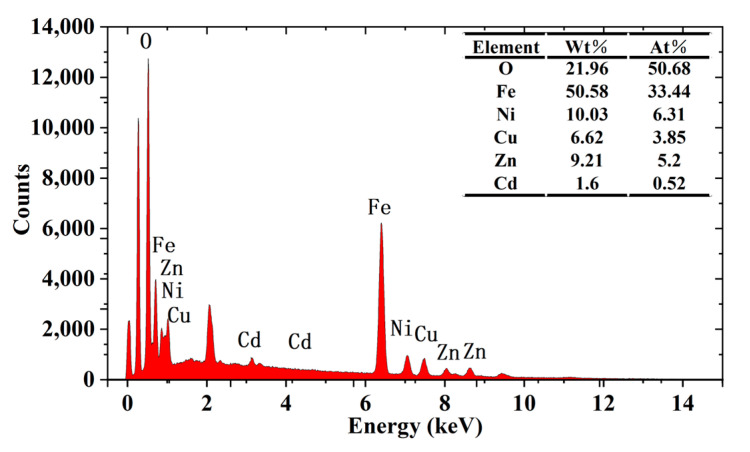
EDA X-ray spectra of a Cd*_x_*Ni_0.5−*x*_Cu_0.2_Zn_0.3_Fe_2_O_4_ sample.

Energy dispersive X-ray spectroscopy (EDX) analysis was conducted on a representative sample where *x* = 0.1 in order to determine the elemental composition of the synthesized samples. Figure 6 indicates the elemental analysis obtained from the EDX analysis of Cd*_x_*Ni_0.5−*x*_Cu_0.2_Zn_0.3_Fe_2_O_4_ nanoparticles. The energy spectrum lines associated with Cd, Ni, Cu, Zn, Fe, and O can be found in the spectra. The values calculated from the spectra for the sample’s elemental composition in atomic and weight percentages are recorded in the embedded table in Figure 6. The results demonstrate that the Cd*_x_*Ni_0.5−_*_x_*Cu_0.2_Zn_0.3_Fe_2_O_4_ sample indicated only the emission peaks of its constituent elements, and no additional impurities were found.

### 2.4. FTIR Analysis

**Figure 7:**
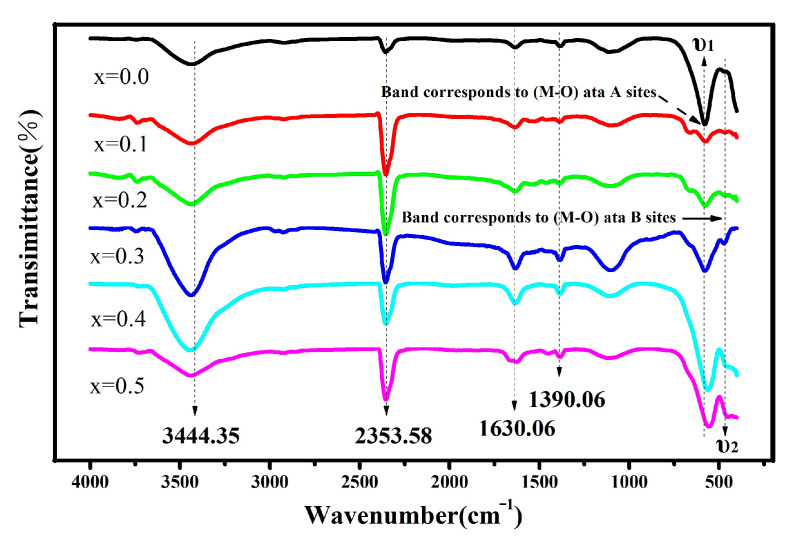
FTIR spectra of Cd*_x_*Ni_0.5−*x*_Cu_0.2_Zn_0.3_Fe_2_O_4_ (0 ≤ *x* ≤ 0.50, in steps of 0.10).

Figure 7 indicates the FTIR spectra of the spinel ferrite system Cd*_x_*Ni_0.5−*x*_Cu_0.2_Zn_0.3_Fe_2_O_4_ (0 ≤ *x* ≤ 0.50, in steps of 0.10). There are two distinct bands between 467 and 580 cm^−1^; the low-frequency absorption band υ_2_ located approximately 460 m^−1^, attributed to stretching vibrations of bonds between tetrahedral (A-site) metal oxides; and the high-frequency absorption band υ_1_ located near 580 cm^−1^, attributed to stretching vibrations of bonds between octahedral (B-site) metal oxides [17,24]. These bands prove the presence of ferrite with spinel structure in the sample [7].

The force constants K_T_ and K_O_ were also calculated from the frequency band data with the following equations [16]:

(8) $$K_{O}=0.942128M{\left(\upsilon_{2}\right)}^{2}/(M+32)$$

(9) $$K_{t}=\sqrt{2}K_{0}\upsilon_{1}/\upsilon_{2}$$

where M is the molecular weight and υ_1_ and υ_2_ are the bands. The frequency bands are inversely related to the bond length. Since υ_1_ is greater than υ_2_, the dimensions of the octahedron (B-site) are larger than those of the tetrahedron (A-site) [3,4]. As shown in Table 4, the range of υ_1_ band decreases as the Cd^2+^ concentration increases, while the υ_2_ band remains constant. This is because the ionic radius of Cd^2+^ is greater than that of Ni^2+^. Moreover, the doping of Cd^2+^ into the tetrahedra increases the Fe–O bond length of the tetrahedra (A-site). On the other hand, the υ_2_ band remains unchanged because non-magnetic divalent Cd^2+^ ions prefer to occupy tetrahedral sites. M.H. Nasr et al. synthesized nanocrystalline spinel ferrites with the general formula Cd_1−*x*_Ni*_x_*Fe_2_O_4_ (0 ≤ *x* ≤ 1.0) using a flash auto-combustion method. With increasing amounts of nickel ions replacing cadmium ions, the υ_1_ and K_T_ values increase. This result is corroborated by the results of the present work [8]. In addition to these two bands, Figure 7 also displays the bands of -NO^3^ ions [9], carboxyl groups [9,11], and hydroxyl groups [4,9,10] appearing at 1390.06, 1630.06, and 3444.35 cm^−1^, respectively.

### 2.5. Dielectric Analysis

**Figure 8:**
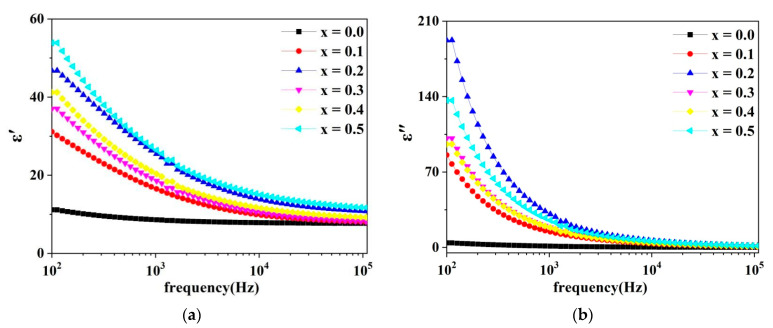
(**a**) Variation of the dielectric constant real part with frequency (**b**) Variation of the dielectric constant imaginary part with frequency.

Figure 8 shows the variation curves of the dielectric constant’s real part of the dielectric constant (ε′), and the imaginary part of the dielectric constant (ε″) with frequency for the spinel ferrite system Cd*_x_*Ni_0.5−*x*_Cu_0.2_Zn_0.3_Fe_2_O_4_ (0.00 ≤ *x* ≤ 0.50 with a step size of 0.10) in the frequency range of 100 Hz to 100 KHz. 

The real part of the dielectric constant is the relative dielectric constant under an electrostatic field, which reflects a material’s ability to store charge. The dielectric constant’s imaginary part represents the energy dissipation that occurs when a dipole in a dielectric overcomes the interference of mechanical collisions under the influence of an electric field, and is oriented back and forth in different directions.

**Figure 9:**
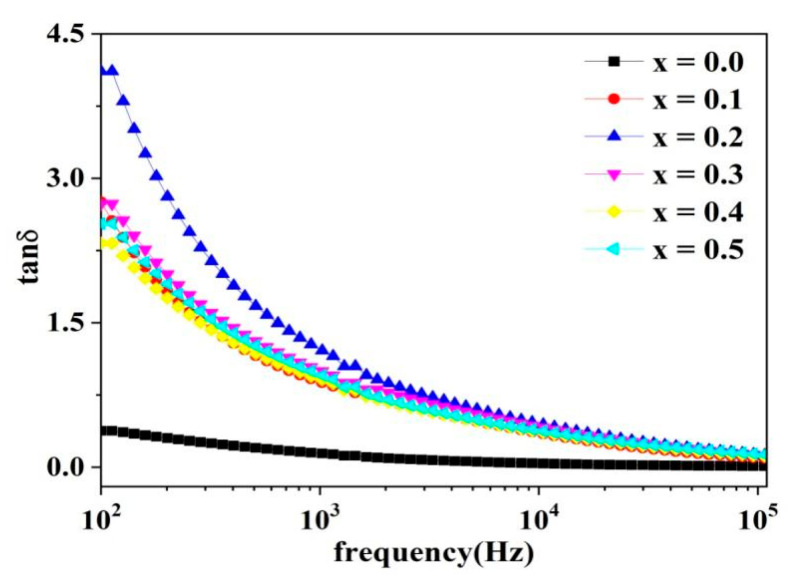
Variation of dielectric loss with frequency.

In dielectric materials, the dielectric loss can be expressed by the loss factor, which is determined by the following equation:

(10) $$\tan\;\delta =\frac{\varepsilon^{\prime \prime }}{\varepsilon^{\prime }}$$

where ε′ is the dielectric constant’s real part, ε″ is the dielectric constant’s imaginary part, and δ is the loss angle. It can be observed in Figure 9 that the dielectric constant real part (ε′) decreases as the frequency increases, and eventually remains constant at high frequencies. The dielectric constant of ferrite depends on the electron transfer mechanism. The electron hopping between Fe^3+^ and Fe^2+^ ions is the primary cause of the ferrite conduction mechanism. Moreover, the hopping electrons have a direct impact on the polarization. The Maxwell–Wagner theory of interfacial polarization provides a detailed explanation for the variation of the dielectric constant with frequency [4,16,17]. According to this polarization theory, grain boundaries with high resistance have a greater impact on the dielectric constant at low frequencies. Moreover, highly conductive grains have a greater impact on the sample at high frequencies. During the annealing treatment of ferrite materials, the irregular distribution of oxygen ions in grains and grain boundaries results in ion and electron displacement polarization at high frequencies, and interfacial polarization at low frequencies. Due to the long response time of interfacial polarization, there is not enough time for response as the frequency increases, resulting in a decrease in the material’s dielectric constant. The dielectric constant eventually maintains a constant value at high frequencies, mainly due to electron displacement polarization. The same results were obtained by M. Moazzam. Hossen et al. in a study using La-doped Ni–Cu–Cd ferrite [4].

The loss factor (tanization theory) and highly resistive grain boundaries have a greater impact on the dielectric constant at low frequencies. The loss factor is high at low frequencies and becomes constant at high frequencies. This is in line with Koop’s theoretical model, which suggests that crystal plane polarization is generated at lower frequencies and that crystal plane polarization primarily contributes to the loss factor at lower frequencies. Due to the long response time of crystal plane polarization, electron transfer and ion exchange at high frequencies cannot be responded to, resulting in a decreasing loss factor with increasing frequency. Ruqayya Zakir., et al. obtained the same results using Ce-doped Co–Mg–Cd ferrites [5].

The real and imaginary parts of the dielectric modulus of the spinel ferrite system Cd*_x_*Ni_0.5−*x*_Cu_0.2_Zn_0.3_Fe_2_O_4_ (0 ≤ *x* ≤ 0.50, in steps of 0.10) are calculated.

(11) $$M^{\prime }=\frac{\varepsilon^{\prime }}{{\varepsilon^{\prime }}^{2}+{\varepsilon^{\prime \prime }}^{2}}$$

(12) $$M^{\prime \prime }=\frac{\varepsilon^{\prime \prime }}{{\varepsilon^{\prime }}^{2}+{\varepsilon^{\prime \prime }}^{2}}$$

**Figure 10:**
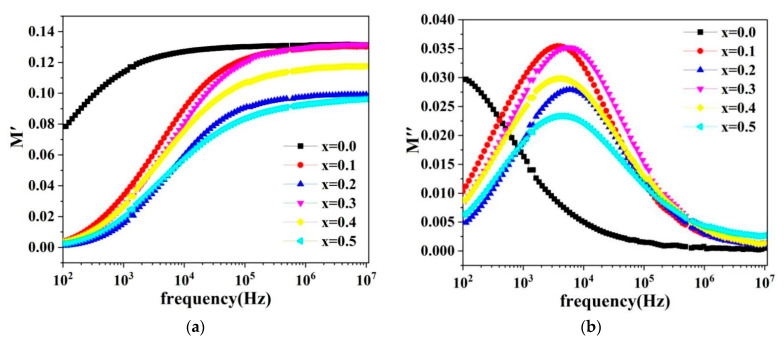
(**a**) Plot of the dielectric modulus real part with frequency (**b**) Plot of the dielectric modulus imaginary part with frequency.

Figure 10a,b indicate the variation curves of the dielectric modulus real part and the dielectric modulus imaginary part with the external field frequency in the frequency range from 100 Hz to 100 KHz. When the frequency is below 10^3^ Hz, the M′ value is close to zero, which is known as the low-frequency region. The M′ value increases continuously with the increase in frequency and reaches the highest point of saturation in the high-frequency region when the frequency is greater than 10^3^ Hz. The spatial variation of the charge carriers (release of space-charge polarization) in the short-range region causes the saturation maxima of M′ in the high-frequency region during the conductivity process. Moreover, the minimum values result from the spatial variation of charge carriers in the long-range region during the conductivity process [4,10]. Figure 10b illustrates that the curve of M″ with frequency has a unique peak (except where *x* = 0.0). The peak changes to higher frequencies as the cadmium content increases. These peaks provide crucial information regarding the type of carrier migration within the system. The peak divides the frequency into two regions: low and high frequencies. In the low-frequency region, the carriers undergo long-range displacement, whereas in the high-frequency region, they experience short-range movement.

**Figure 11:**
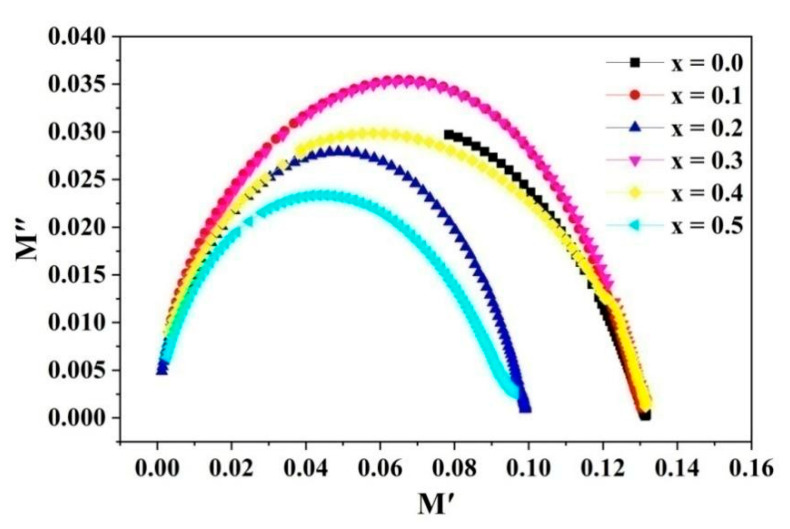
Nyquist plot of Cd*_x_*Ni_0.5−*x*_Cu_0.2_Zn_0.3_Fe_2_O_4_ (0 ≤ *x* ≤ 0.50, in steps of 0.10).

The sample’s Nyquist plot is shown in Figure 11. Evenly curving arcs can be observed in the figure (except for where *x* = 0.0). This indicates that grain boundaries occupy a larger volume and that grain boundaries dominate the conductive behavior [16]. By analyzing the complex modulus, it is possible to study materials with the same resistance but different capacitances. M.M. Hossen et al. [4,10] obtained dielectric experimental results using La and Mn -doped Ni–Cu–Cd ferrites, which are in agreement with our experimental results.

### 2.6. Magnetic Properties

**Figure 12:**
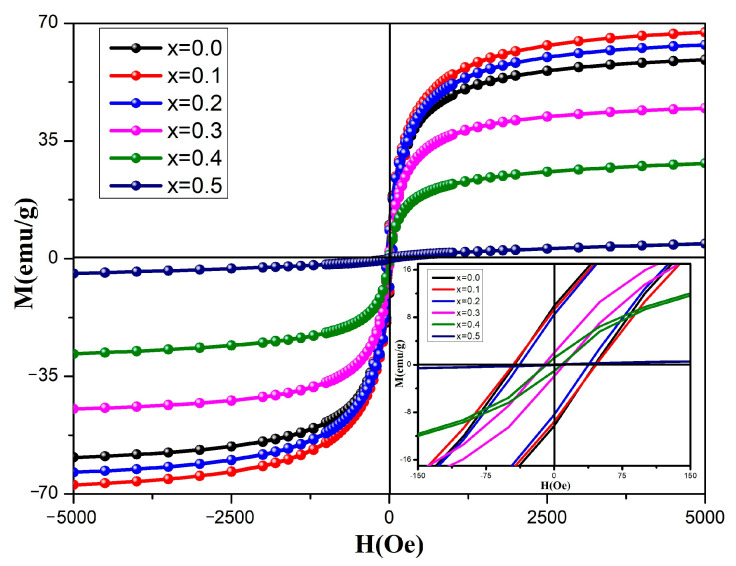
Hysteresis lines of Cd^2+^-doped Ni–Cu–Zn spinel ferrites. Hysteresis loops from −150 Oe to 150 Oe are magnified in the lower right inset.

Figure 12 depicts the measured hysteresis lines of the spinel ferrite system Cd*_x_*Ni_0.5−*x*_Cu_0.2_Zn_0.3_Fe_2_O_4_ (0 ≤ *x* ≤ 0.50, in steps of 0.10) at room temperature. The sample hysteresis line is S-shaped when *x* = 0.0–0.4, indicating that the sample is ferromagnetic. The hysteresis line of the *x* = 0.5 sample is close to a straight line, indicating that the sample is weakly ferromagnetic and close to being paramagnetic. 

Hysteresis lines were used to estimate the values of saturation magnetization strength (M_s_), coercivity (H_c_), residual magnetization strength (M_r_), residual squareness (M_r_/M_s_), magnetic moment (μ_B_), and each anisotropy (K_1_). All magnetic parameters are reported in Table 5. Evidently, the saturation magnetization intensity of the Cd*_x_*Ni_0.5−*x*_Cu_0.2_Zn_0.3_Fe_2_O_4_ sample increases initially and then decreases with increasing Cd content. The saturation magnetization intensity of ferrite is dependent on the distribution of magnetic cations in the A and B sites, the sintering temperature, grain size, chemical composition, density, etc. The saturation magnetization strength of ferrite depends strongly on the exchange interaction between the A- and B-site cations [10,17]. The intensity of the exchange interactions in a ferrite system is proportional to the distance between the cations. Therefore, ion-doped ferrite with different particle sizes can alter the ferrite’s magnetization strength. The magnetic variation of ferrite can be explained by Neel theory. Based on Neel theory, the M_s_ of spinel-type ferrite can be expressed by the following equation:

(13) $$M_{s}=\left|M_{B}-M_{A}\right|$$

where M_A_ and M_B_ are the magnetization intensities of the A- and B-site ions, respectively.

The increase and then decrease in magnetization intensity of the sample with increasing Cd^2+^ content may be a result of the tendency of non-magnetic Cd^2+^ ions to occupy the A-site (M_A_ decreases), causing the Fe^3+^ ions at the A-site to migrate to the B-site after being compressed (M_B_ increases). This, based on Neel’s theory, increases the sample’s magnetization intensity. The subsequent decrease in the samples’ magnetization intensity may be a result of the production of the paramagnetic material α-Fe_2_O_3_ in the sample. The magnetic moment for each formula unit in the Bohr magnetron is obtained using the following equation [8]:

(14) $$\mu_{B}=\frac{M_{s}\times M_{W}}{5585}$$

where M_s_ is the saturation magnetization intensity and M_W_ is the sample’s molecular weight. In a Bohr magnetron, the trend of the magnetic moment for each formula unit is the same as the saturation magnetization intensity. The same phenomenon was discovered by Kaur, Harpreet, et al. using non-magnetic ions in Cd-doped CoFe_2_O_4_ [25]. The coercivity of the samples decreases as the Cd^2+^ increases, as can be observed from the lower right inset of Figure 12. The variation of coercivity is determined by various factors, such as particle size, crystallinity, crystal structure, morphology, strain, and anisotropy [24,26]. Based on the Stoner Wolfarth findings, values for the theoretical squareness (M_r_/M_s_) below 0.5 indicate that the nanoparticles are uniaxially anisotropic, single-domain materials [3,6,24]. The squareness of the samples (M_r_/M_s_) falls in the range of 0.0120–0.1672, indicating that the material is anisotropic and single-domain. Table 5 illustrates the various anisotropies [3,4,10], where M_s_ is the saturation magnetization intensity and H_c_ is the coercivity [8]. The Cd*_x_*Ni_0.5−*x*_Cu_0.2_Zn_0.3_Fe_2_O_4_ sample has excellent hysteresis lines and low coercivity, indicating that the sample is easily demagnetized and has the potential to be an excellent electromagnet material [11,14].

## 3. Experimental Section

**Figure 13:**
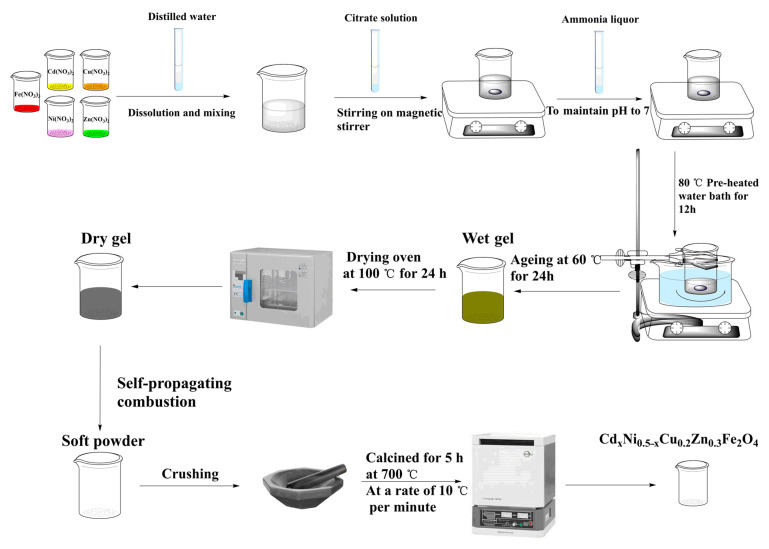
Process flow diagram of a Cd^2+^-doped Ni–Cu–Zn spinel ferrite sample.

The sol–gel method was used to prepare cadmium-doped Ni–Cu–Zn ferrite (Figure 13). The general formulation was Cd*_x_*Ni_0.5−*x*_Cu_0.2_Zn_0.3_Fe_2_O_4_ (*x* = 0.10, 0.20, 0.30, 0.40, 0.50). The materials were purchased from West Long Chemical; namely, cadmium nitrate tetrahydrate (Cd(NO_3_)_2_·4H_2_O), nickel nitrate hexahydrate (Ni(NO_3_)_2_·6H_2_O), copper nitrate trihydrate (Cu(NO_3_)_2_·3H_2_O), zinc nitrate hexahydrate (Zn(NO_3_)_2_·6H_2_O), iron nitrate nonahydrate (Fe(NO_3_)_3_·9H_2_O), ammonium hydroxide (NH_3_·H_2_O), and mono citric acid hydrate (C_6_H_10_O_8_). All metal nitrates were weighed based on the stoichiometric ratio and dissolved in distilled water, and then the solution was stirred using a magnetic stirrer and stirred evenly to ensure complete dissolution. Citric acid was added at a ratio of 1:1 (nitrate to citric acid), and the solution continued to be stirred at a constant rate. To create a pH = 7 precursor solution, ammonia was added dropwise at room temperature. The precursor solution was heated in a water bath at 80 °C while being constantly stirred. Eventually, the solution became a wet gel, which was then baked in a drying oven at 100 °C for 24 h until completely dry. Finally, the soft powder-like material was obtained by self-propagation. The same method was used to prepare each sample. The samples were then crushed and calcined for 5 h at 700 °C at a rate of 10 °C per minute to create extremely crystalline samples. XRD analysis was performed on an X’Pert3 powder-type multifunctional X-ray diffractometer. Cu Kα was utilized as the radiation source (λ = 0.15406 nm), and the scanning range was 20–80°. The crystal structure and lattice parameters of the samples were determined using the Fullprof and Rietveld methods. The infrared spectra of the samples were measured using a NICOLET 6700 Fourier transform infrared spectrometer with a scan range of 450–4000 Hz. The indicated morphology and microstructure of the samples were examined using a TASKEN MIRALMS scanning electron microscope (SEM) with an inbuilt Oxford Xplore 30 energy spectrometer (EDS). The hysteresis lines of the samples were measured using the Magnetic Property Measurement System (MPMS3) at room temperature, with a maximum applied magnetic field of 5 kOe. Frequency-dependent dielectric spectra were obtained using a WayneKer impedance analyzer (model 6500P) in the range of 100 Hz to 100 kHz at room temperature.

## 4. Conclusions

Using the sol–gel self-propagation method, Cd*_x_*Ni_0.5−*x*_Cu_0.2_Zn_0.3_Fe_2_O_4_ (0 ≤ *x* ≤ 0.50) spinel-type ferrite was prepared using the sol–gel self-propagation technique in this study. In addition, XRD analysis indicated that the samples formed a space group of Fd-3m cubic spinel structure ferrite, while the second phase α-Fe_2_O_3_ appeared in the samples with Cd doping, indicated that the solid solution limit of cadmium-doped Ni–Cu–Zn ferrite was less than *x* = 0.1. The lattice constant of the samples increased from 8.3794 Å to 8.5397 Å with the continuous increase of the Cd content, and the increase of lattice constants The increase in lattice constant is due to the larger ionic radius of Cd (0.97 Å) than that of Ni (0.69 Å). FTIR analysis determined two distinct bands between 467 and 580 cm^−1^, corresponding to the stretching vibrations of the bonds between tetrahedral and octahedral metal oxides. This results in the band υ1 decreasing from 580.50 cm^−1^ to 556.23 cm^−1^, and the tetrahedral force constant decreasing from 3.1880 × 10^2^ N/m to 3.0921 × 10^2^ N/m caused by the preference of Cd^2+^ ions to occupy the tetrahedral sites. The decrease in the sample dielectric property parameters with increasing frequency can be explained in detail by Maxwell–Wagner interface polarization theory, where the high-frequency dielectric constant remains constant at low values resulting from the major role of electron displacement polarization. Magnetic analysis shows that the samples are ferromagnetic when *x* = 0.0–0.4, and weakly ferromagnetic and nearly paramagnetic when *x* = 0.5. The saturation magnetization intensity of the samples increases with the increase of Cd content to 67.2958 emu/g (*x* = 0.1) and then decreases rapidly to 4.4228 emu/g (*x* = 0.5). The hysteresis return lines of Cd*_x_*Ni_0.5−*x*_Cu_0.2_Zn_0.3_Fe_2_O_4_ (0.0 ≤ *x* ≤ 0.4) samples are good, with low coercivity and squareness. These nanoparticles are easy to demagnetize and may be suitable for magnetic recording and soft magnetic ferrite applications, including biomedical applications (e.g., thermal therapy).

## Figures and Tables

**Table 1:** Structural parameters of Cd^2+^-doped Ni–Cu–Zn spinel ferrite nanomaterials.

Parameters	Concentration (*x*)					
	0	0.10	0.20	0.30	0.40	0.50
2θ° (311) peak	35.4860	35.3720	35.7270	35.1100	34.9870	34.7880
d (Å)	0.2529	0.2536	0.2542	0.2554	0.2563	0.2577
a = b = c	8.3794	8.3987	8.4197	8.4668	8.4901	8.5397
D (nm)	55.02	52.52	62.18	56.83	53.95	49.52
V (Å)^3^	588.3625	592.4331	596.8860	606.9505	611.9946	622.7746
ρ_x_ (g·cm^−3^)	5.3594	5.4420	5.5219	5.5479	5.6188	5.6362
ε	6.27 × 10^−4^	3.72 × 10^−4^	1.18 × 10^−3^	2.22 × 10^−3^	1.46 × 10^−3^	1.84 × 10^−3^

**Table 2:** Rietveld refinement parameters of Cd^2+^-doped Ni–Cu–Zn spinel ferrite nanomaterials.

*x*	R-Factors			GoF	Lattice Parameters		
	R_p_	R_wp_	R_exp_	χ^2^	a_rv_ (Å)	V (Å)^3^	Fract (%)
0.0	0.66	0.82	0.74	1.28	8.378	588.030	100
0.1	0.67	0.87	0.74	1.40	8.394	591.417	97.93
0.2	0.73	0.92	0.76	1.47	8.420	596.876	96.28
0.3	0.81	1.04	0.80	1.68	8.462	605.924	96.33
0.4	0.79	1.07	0.81	1.75	8.485	610.950	91.67
0.5	0.89	1.14	0.87	1.72	8.532	621.066	91.98

**Table 3:** Bond lengths of Cd^2+^-doped Ni–Cu–Zn spinel ferrite nanomaterials.

*x*	L_A_	L_B_	A-O	B-O	r_A_	r_B_
0.0	3.628	2.962	1.9299	2.0297	0.6099	0.7097
0.1	3.635	2.968	1.9336	2.0335	0.6136	0.7135
0.2	3.646	2.977	1.9396	2.0398	0.6196	0.7198
0.3	3.664	2.992	1.9493	2.0500	0.6293	0.7300
0.4	3.674	3.000	1.9545	2.0555	0.6345	0.7355
0.5	3.694	3.016	1.9654	2.0670	0.6454	0.7470

**Table 4:** FTIR parameters of Cd^2+^-doped Ni–Cu–Zn spinel ferrite nanomaterials.

Cd^2+^	υ_1_	K_T_	υ_2_	K_O_
Content, *x*	(cm^−1^)	(dyne/cm) × 10^5^	(cm^−1^)	(dyne/cm) × 10^5^
0.0	580.50	3.1880	467.76	1.8164
0.1	580.50	3.1964	467.76	1.8212
0.2	580.50	3.2045	467.76	1.8258
0.3	580.50	3.2123	467.76	1.8303
0.4	566.41	3.1416	467.76	1.8346
0.5	556.23	3.0921	467.76	1.8387

**Table 5:** Magnetic parameters of Cd^2+^-doped Ni–Cu–Zn spinel ferrites.

Cd^2+^ Content, *x*	M_s_ (emu/gm)	M_r_ (emu/gm)	M_r_/M_s_	Coercivity (Oe)	μ_B_ (Bohr Magneton)	K_1_ = M_s_H_c_/ 2 × sH^4^ (erg/cm^3^)
0.0	59.1433	9.8904	0.1672	45.1505	2.5136	0.1335
0.1	67.2958	9.4579	0.1405	46.1747	2.9248	0.1554
0.2	63.5720	8.4354	0.1327	38.3361	2.8241	0.1219
0.3	47.7300	1.9719	0.0413	10.8696	2.1662	0.0259
0.4	28.3063	1.0590	0.0374	7.3370	1.3119	0.0104
0.5	4.4228	0.0530	0.0120	9.5443	0.2092	021

## Data Availability

Not applicable.

## Sample Availability

Samples of the compounds are available from the authors.
